# Switching from entecavir to tenofovir disoproxil fumarate for HBeAg-positive chronic hepatitis B patients: a phase 4, prospective study

**DOI:** 10.1186/s12876-021-02008-9

**Published:** 2021-12-20

**Authors:** Fumitaka Suzuki, Yoshiyuki Suzuki, Yoshiyasu Karino, Yasuhito Tanaka, Masayuki Kurosaki, Hiroshi Yatsuhashi, Tomofumi Atarashi, Masanori Atsukawa, Tsunamasa Watanabe, Masaru Enomoto, Masatoshi Kudo, Naoto Maeda, Hiroshi Kohno, Kouji Joko, Kojiro Michitaka, Koichiro Miki, Kazuhiro Takahashi, Tatsuya Ide, Shigetoshi Fujiyama, Tomoko Kohno, Hiroshi Itoh, Sakiyo Tsukamoto, Yuko Suzuki, Yoshiaki Kawano, Wataru Sugiura, Hiromitsu Kumada

**Affiliations:** 1 Toranomon Hospital Kajigaya, 1-3-1, Kajigaya, Takatsu-ku, Kawasaki-city, Kanagawa 213-8587 Japan; 2 Toranomon Hospital, 2-2-2, Toranomon, Minato-ku, 105-8470 Japan; 3 Sapporo-Kosei General Hospital, 8-5, Kita 3-jo Higashi, Chuo-ku, Sapporo-city, Hokkaido 060-0033 Japan; 4 Nagoya City University Hospital, 1, Aza-Kawasumi, Mizuho, Nagoya, Aichi 467-8602 Japan; 5 Musashino Red Cross Hospital, 1-26-1, Kyonan-cho, Musashino-shi, Tokyo, 180-8610 Japan; 6 National Hospital Organization Nagasaki Medical Center, 2-1001-1, Kubara, Omura-city, Nagasaki 856-8562 Japan; 7 Obihiro-Kosei General Hospital, 10-1, Nishi 14-jo Minami, Obihiro-city, Hokkaido 080-0024 Japan; 8 Nippon Medical School Chiba Hokusoh Hospital, 1715, Kamakari, Inzai-City, Chiba 270-1694 Japan; 9 St. Marianna University School of Medicine Hospital, 2-16-1, Sugao, Miyamae-ku, Kawasaki-city, Kanagawa 216-8511 Japan; 10 Osaka City University Hospital, 1-5-7, Asahi-machi, Abeno-ku, Osaka-city, Osaka 545-8586 Japan; 11 Kindai University Hospital, 377-2, Ohnohigashi, Osakasayama-city, Osaka 589-8511 Japan; 12 Sanin Rosai Hospital, 1-8-1, Kaikeshinden, Yonago-city, Tottori 683-8605 Japan; 13 National Hospital Organization Kure Medical Center and Chugoku Cancer Center, 3-1, Aoyama-cho, Kure-city, Hiroshima 737-0023 Japan; 14 Matsuyama Red Cross Hospital, 1, Bunkyo-cho, Matsuyama-city, Ehime 790-8524 Japan; 15 Ehime Prefectural Central Hospital, 83, Kasugamachi, Matsuyama-city, Ehime 790-0024 Japan; 16 Kitakyushu City Hospital Organization Kitakyushu Municipal Medical Center, 2-1-1, Bashaku, Kokurakita-ku, Kitakyushu-city, Fukuoka 802-0077 Japan; 17 Hamanomachi Hospital, 3-3-1, Nagahama, Chuo-ku, Fukuoka-city, Fukuoka 810-8539 Japan; 18 Kurume University Hospital, 67, Asahi-machi, Kurume-shi, Fukuoka, 830-0011 Japan; 19 Kumamoto Shinto General Hospital, 3-2-65, Ooe, Chuo-ku, Kumamoto-city, Kumamoto 862-8655 Japan; 20 GlaxoSmithKline K.K., Akasaka Intercity AIR, 1-8-1, Akasaka, Minato-ku, Tokyo, 107-0052 Japan; 21 National Center for Global Health and Medicine, 1-21-1 Toyama Shinjuku-ku, Tokyo, 162-8655 Japan; 22 Present Address: Keiyukai Sapporo Hospital, 1-1, Kita, Hondori 14 chome, Shiroishi-ku, Sapporo-city, Hokkaido 003-0027 Japan; 23 Present Address: Kumamoto University, 1-1-1 Honjo, Chuo-ku, Kumamoto, 860-8556 Japan; 24 Present Address: Shin-Eikai Hospital, 12-11, Bentencho, Kokurakita-ku, Kitakyushu-city, Fukuoka 803-0856 Japan

**Keywords:** Tenofovir disoproxil fumarate, Chronic hepatitis B, HBsAg, HBeAg-positive, Entecavir

## Abstract

**Background:**

Tenofovir disoproxil fumarate (TDF) is widely used and recommended as first-line treatment for patients infected with the hepatitis B virus (HBV). However, current data are limited regarding the efficacy and safety of switching to TDF for the treatment of chronic hepatitis B in hepatitis B e-antigen (HBeAg)-positive patients who are virologically suppressed with another nucleos(t)ide analogue. The primary objective of this study was to evaluate the hepatitis B surface antigen (HBsAg) reduction potential of switching from entecavir (ETV) to TDF at week 48 in HBeAg-positive chronic hepatitis B patients with undetectable serum HBV-DNA.

**Methods:**

In this multicenter, single-arm, open-label, phase 4 clinical study, 75 participants currently treated with ETV 0.5 mg once daily were switched to TDF 300 mg once daily for 96 weeks.

**Results:**

At week 48, 3/74 participants (4%) achieved 0.25 log_10_ reduction of HBsAg levels from baseline (the primary endpoint). Mean HBsAg reduction was −0.14 log_10_ IU/mL and 12% (9/74) achieved 0.25 log_10_ reduction by 96 weeks. No participants achieved HBsAg seroclearance. HBsAg reduction at weeks 48 and 96 was numerically greater in participants with higher alanine aminotransferase levels (≥ 60 U/L). Seventeen participants (25%) achieved HBeAg seroclearance up to week 96. No participants experienced viral breakthrough. All drug-related adverse events (18 participants [24%]) were mild in intensity, including an increase in urine beta-2-microglobulin (15 participants [20%]).

**Conclusions:**

In conclusion, HBsAg reduction was limited after switching from ETV to TDF in this study population. Further investigation is warranted to better understand the clinical impact of switching from ETV to TDF.

*ClinicalTrials.gov*: NCT03258710 registered August 21, 2017. https://clinicaltrials.gov/ct2/show/NCT03258710?term=NCT03258710&draw=2&rank=1

## Background

Hepatitis B virus (HBV) infection is characterized by the serologic presence of hepatitis B e-antigen (HBeAg) and HBV-DNA, as well as hepatitis B surface antigen (HBsAg) [1]. Oral nucleos(t)ide analogues (NAs) such as lamivudine, entecavir (ETV), tenofovir alafenamide (TAF), and tenofovir disoproxil fumarate (TDF) effectively inhibit reverse transcription of pregenomic HBV-RNA, thereby lowering serum HBV-DNA levels [2, 3]. Achieving HBsAg seroclearance is associated with sustained immunologic and virologic control by appropriate treatments and a reduced risk of hepatocellular carcinoma (HCC) [4–6]. While the long-term treatment goal of HBsAg seroclearance is rarely achievable with current treatment options, including NAs [7, 8], several clinical trials have been conducted to seek better treatment options to approach HBsAg seroclearance [9–11].

Regarding HCC risk during treatment with NAs, a study in Korea indicated that treatment with TDF has been associated with a significantly lower risk of HCC compared with ETV in chronic hepatitis B (CHB) patients [12]. Moreover, previous studies in CHB patients conducted by our group demonstrated superior HBsAg reduction with TDF compared with other NAs in NA-naïve patients. In addition, reduction in HBsAg was previously achieved predominantly in HBeAg-positive patients with relatively high viral activity who were receiving TDF [4, 13].

Previous studies, mainly in HBeAg negative patients, have reported short-term efficacy after switching from ETV to TDF [11, 14]. However, current data are limited, focusing on HBeAg-positive patients with CHB who are virologically suppressed with another NA. Furthermore, the impact of patient factors on the reduction of HBsAg remains unclear [13, 15]. Based on previous studies showing a decline of HBsAg in patients with HBeAg-positive CHB and undetectable serum HBV-DNA who were treated with ETV therapy, we predicted that HBsAg reduction after switching from ETV to TDF could potentially be of clinical benefit.

## Methods

### Study design

This was a multicenter, single-arm, open-label clinical study conducted in HBeAg-positive CHB patients with undetectable serum HBV-DNA (initiated October 2, 2017 and completed November 25, 2019). Participants (N = 75) currently treated with ETV 0.5 mg once daily were switched to TDF 300 mg on day 1, following a 6-week screening period. TDF was administered once daily for 96 weeks.

The study was approved by the ethics committee at every participating institution (19 centers in Japan) and was conducted according to the recommendations of Good Clinical Practice and the Declaration of Helsinki (2013). All participants provided written informed consent to participate in the study.

### Study participants

Male and female patients with CHB between 20 and 69 years of age at the time of informed consent who had been treated with ETV for at least two years were eligible for study inclusion. At screening, participants were required to test positive for serum HBeAg (local hospital or central laboratory), have an HBV-DNA level below the limit of quantitation (< 1.3 log_10_ IU/mL [< 20 IU/mL]), and have a serum HBsAg level ≥ 800 IU/mL (or 80 to 800 IU/mL and fluctuation decrease within 0.1 log_10_ IU/mL per year to exclude participants with continuous HBsAg decrease). Additional inclusion criteria included creatinine clearance ≥ 70 mL/min, hemoglobin ≥ 8 g/dL, and white blood cell count ≥ 1000/mm^3^.

Patients who received any interferon (IFN) or HBV vaccine therapy within 24 weeks prior to initiation of the study treatment; or TDF, adefovir dipivoxil (ADV), or TAF within two years prior were excluded. Patients coinfected with HIV or hepatitis C virus (HCV) were excluded. Patients with a history of (or suspected of having) HCC were also ineligible. Other exclusion criteria included drugs causing renal impairment, competitors of renal excretion, immunosuppressants, or glucocorticoids within eight weeks prior to study initiation; the presence of proximal tubulopathy; decompensated CHB (direct bilirubin > 1.5 × the upper limit of normal; prothrombin time < 60%; platelets < 75,000/mm^3^; albumin < 3.0 g/dL); or a history of alcohol or drug abuse.

### Clinical evaluations

Virologic testing at the time of screening included HIV, HCV, HBV genotype, HBV-DNA levels, HBeAg/anti-HBe, and HBsAg/anti-HBs quantitation. HBV-DNA, HBeAg/anti-HBe, HBsAg/anti-HBs, and hepatitis B core-related antigen (HBcrAg) testing was performed at week 4, week 12, and every 12 weeks thereafter, by SRL Medisearch Inc. (Tokyo, Japan). Dual energy X-ray absorptiometry (DXA) testing was performed at screening, last visit, and at intervals as needed based on laboratory results, but not more frequently than every 4 months.

### Study endpoints

The primary endpoint of this study was the proportion of participants achieving 0.25 log_10_ HBsAg reduction from baseline at week 48 (defined as the HBsAg responder rate). The secondary endpoint was the proportion of participants achieving 0.25 log_10_ HBsAg reduction from baseline at weeks 24 and 96. Additional secondary efficacy analyses at weeks 24, 48, and 96 included the proportion of participants achieving HBsAg seroclearance and HBsAg/anti-HBs seroconversion, reduction of HBsAg from baseline, the proportion of participants achieving HBeAg seroclearance and HBeAg/anti-HBe seroconversion, and reduction of HBcrAg from baseline.

Safety assessments included the monitoring of adverse events (AEs), as well as urinalysis, hematology, and clinical chemistry testing, which was performed at baseline and again at weeks 4 and 12, and every 12 weeks thereafter. Adverse events were summarized as study drug-related AEs, AEs by intensity, and those resulting in discontinuation of the study. Adverse events of special interest (AESIs) included those with renal, liver, or bone involvement.

### Statistical analyses

Descriptive statistics were used to assess the efficacy and safety objectives. An estimation approach was used to address the efficacy objectives, where point estimates and corresponding 95% confidence intervals (CIs) were constructed.

Although no confirmatory hypotheses were tested in this study, the sample size was based on the expected responder rate and threshold rate. Results from the previous Japanese phase 3 study [13] showed that the proportion of participants expected to achieve 0.25 log_10_ HBsAg reduction from the baseline at week 48 was 20%. Additionally, a 0.25 log_10_ HBsAg reduction after 48 weeks of TDF therapy was considered to be clinically meaningful and defined as a primary endpoint in this study. The proportion of HBsAg responders in subjects not switched to TDF-based regimens on the basis of results of ETV treatment was assumed to be 6% (threshold rate), and the sample size with at least 90% power to detect a 14% difference against the threshold was calculated to be 57. Allowing for a participant dropout rate of 10%, the target sample size was set at approximately 65.

Analyses of safety data were performed at screening, baseline, weeks 4, 12, 24, 36, 48 and week 96 using the safety population, defined as all participants who received study treatment. Analyses of efficacy data were performed at screening, baseline, weeks 4, 12, 24, 36, 48, and week 96 using the full analysis set (FAS), defined as all participants enrolled who received study treatment and who had efficacy data available at least 15 days after starting treatment. A subset of participants, which excluded three participants in the FAS who met the protocol deviation criteria of receiving medication, was defined as the efficacy evaluable set (EES). Analysis of the EES was performed to evaluate the robustness of the efficacy results.

## Results

**Fig. 1:**
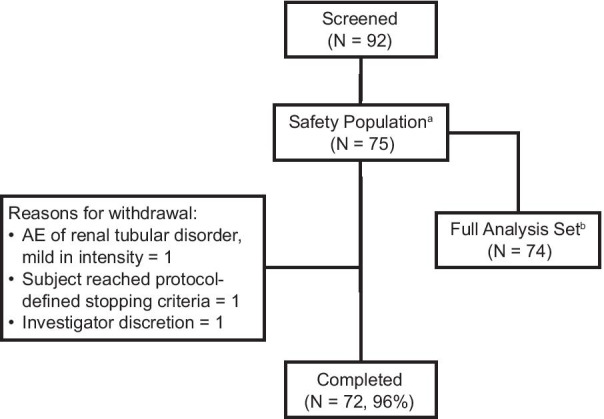
Participant disposition. AE, adverse event. ^a^Participants who received at least 1 dose of study treatment after enrollment. ^b^Excludes participants who had no efficacy data at least 15 days after the start of study treatment

A total of 92 patients were screened and 75 patients enrolled in the study (safety population) (Fig. 1).

The FAS included 74 participants, 72 of whom completed week 96. One participant withdrew from the study due to a study drug-related adverse event, one participant reached protocol-defined liver stopping criteria (bilirubin ≥ 2 × the upper limit of normal and direct bilirubin > 35%) on day 1 before receiving TDF, and one participant was withdrawn at the discretion of the investigator.

### Participant characteristics

Of the 75 participants (SP), 70 were HBeAg-positive at baseline. Another five participants who were HBeAg-positive at screening according to local in-hospital laboratory results were HBeAg-negative at screening and baseline according to central laboratory results. For all participants, serum HBV-DNA level was undetectable (< 1.3 log_10_ IU/mL [< 20 IU/mL]) at baseline except for one participant with 1.9 log_10_ IU/mL. No patients met protocol-defined criteria for liver cirrhosis at baseline. Baseline characteristics for the safety population are shown in Table 1.

**Table 1:** Participant demographics and baseline characteristics (SP)

Baseline characteristic	TDF (N = 75)
Age (years), mean (± SD)	48.4 (± 9.35)
Sex, n (%)	
Male	55 (73)
Female	20 (27)
Race, n (%)	
Japanese	71 (95)
East Asian	4 (5)
HBV genotype, n (%)	
A	1 (1)
B	2 (3)
C	72 (96)
HBV-DNA (log_10_ IU/mL), mean (± SD)	0.92 (± 0.12)
ALT (IU/L), mean (± SD)	23.5 (± 18.13)
HBeAg, n (%)	
Positive	70 (93)
Negative^a^	5 (7)
HBsAg (IU/mL), mean (± SD)	5311.3 (± 5619.84)
HBcrAg (log_10_ U/mL), mean (± SD)	5.5 (± 0.55)
eGFR by JSN-CKDI (mL/min/1.73m^2^), n (%)	
< 60	0 (0)
60 to < 90	42 (56)
≥ 90	33 (44)
Urine beta-2-microglobulin (µg/g Cr), mean (± SD)	241.9 (305.58)
Protocol defined liver cirrhosis, n (%)	
Yes	0 (0)
No	75 (100)

*ALT* alanine aminotransferase, *Cr* creatinine, *eGFR by JSN-CKDI* estimated glomerular filtration rate calculated by the Japanese Society of Nephrology-Chronic Kidney Disease Initiatives equation, *HBV* hepatitis B virus, *HBeAg* hepatitis B e-antigen, *HBsAg* hepatitis B surface antigen, *HBcrAg* hepatitis B core-related antigen, *SD* standard deviation, *SP* safety population, *TDF* tenofovir disoproxil fumarate

^a^HBeAg-positive according to local in-hospital laboratory results, but HBeAg-negative according to central laboratory results at screening

### Antiviral responses

The proportion of participants achieving 0.25 log_10_ HBsAg reduction from baseline at week 48 was 3/74 (4%) participants (Table 2). All three participants were male, infected with HBV genotype C, and were similar in age (45, 44, and 42 years). One of these participants was HBeAg-negative at baseline but was judged HBeAg-positive according to a local laboratory at screening. At 96 weeks, the proportion of participants achieving > 0.25 log_10_ reduction from baseline was 12% (9/74, including the three participants who had responded by week 48).

**Table 2:** Antiviral responses after 48 and 96 weeks of treatment (FAS)

Key efficacy endpoints	Baseline	Week 48	Week 96
Positive HBsAg at baseline (N = 74)			
Participants achieving 0.25 log_10_ HBsAg reduction from baseline			
HBsAg ≤ −0.25 log_10_, n (%), [95% CI]	–	3 (4), [0.8, 11.4]	9 (12), [5.7, 21.8]
Participants achieving HBsAg seroclearance		0	0
Participants achieving HBsAg/anti-HBs seroconversion^a^		0	0
Change from baseline in HBsAg (log_10_ IU/mL)			
Mean (SD), [95% CI]	3.52 (0.46), [3.41, 3.62]^b^	−0.11 (0.08), [−0.13, −0.10]^c^	−0.14 (0.12), [−0.17, −0.11]^a^
Positive HBeAg at baseline (N = 69)			
Participants achieving HBeAg seroclearance, n (%)		8 (12)	17 (25)
Participants achieving HBeAg/anti-HBe seroconversion,^d^ n (%)		7 (13)	13 (25)
Change from baseline in HBcrAg (N = 74), (log_10_ U/mL)			
Mean (SD), [95% CI]	5.53 (0.54), [5.40, 5.65]^b^	−0.17 (0.29), [−0.23, −0.10]^c^	−0.30 (0.36), [−0.38, −0.22]^a^
Change from baseline in HBV-DNA (N = 74), (log_10_ IU/mL)			
Mean (SD), [95% CI]	0.92 (0.12), [0.89, 0.95]^b^	−0.02 (0.10), [−0.04, 0.01]^c^	−0.02 (± 0.12), [−0.05, 0.01]^a^

*Anti-HBe* anti-hepatitis B e-antibody, *FAS* full analysis set, *Anti-HBs* hepatitis B surface antibody, *CI* confidence interval, *HBeAg* hepatitis B e-antigen, *HBcrAg* hepatitis B core-related antigen, *HBsAg* hepatitis B surface antigen, *HBV* hepatitis B virus, *SD* standard deviation, *TDF* tenofovir disoproxil fumarate

^a^The mean and SD baseline values are absolute values

^b^N = 74

^c^N = 73

^d^HBeAg/Ab seroconversion was analyzed in the 53 participants who were HBeAg-positive and anti-HBe-negative at baseline

**Fig. 2:**
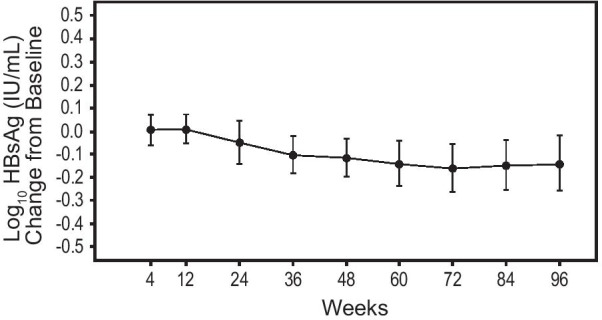
Mean change in HBsAg up to week 96 (FAS). FAS, full analysis set; HBsAg, hepatitis B surface antigen

The overall mean (± standard deviation [SD]) change from baseline in HBsAg level decreased, with a mean change of −0.11 (± 0.08) log_10_ IU/mL at week 48 and −0.14 (± 0.12) log_10_ IU/mL at week 96 (Table 2 and Fig. 2).

**Fig. 3:**
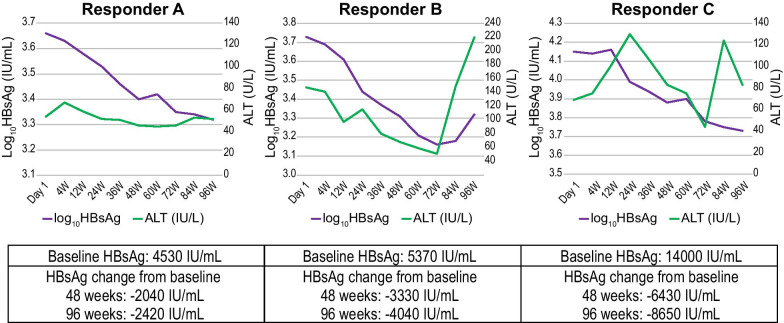
Individual Responder Change in HBsAg and ALT (FAS). ALT, alanine aminotransferase; FAS, full analysis set; HBsAg, hepatitis B surface antigen; W, weeks

At weeks 48 and 96, HBsAg reduction from baseline was numerically greater in the higher alanine aminotransferase (ALT) group (defined as ≥ 60 U/L at least once from baseline) compared with the lower ALT group (defined as < 60 U/L from baseline). Mean (± SD; [95% CI]) change in HBsAg from baseline at week 48 was −0.25 (± 0.09; [−0.35, −0.15]) log_10_ IU/mL in the higher ALT group (N = 6), versus −0.10 (± 0.07; [−0.12, −0.08]) log_10_ IU/mL in the lower ALT group (N = 67). At week 96, mean change from baseline in HBsAg was −0.37 (± 0.14; [−0.52, −0.22]) in the higher ALT group (N = 6) and −0.12 (± 0.09; [−0.14, −0.09]) in the lower ALT group (N = 66). All three participants who achieved 0.25 log_10_ HBsAg reduction from baseline at week 48 demonstrated high serum ALT levels throughout the study period as shown in Fig. 3.

Of the additional six participants who achieved 0.25 log_10_ HBsAg reduction from baseline by week 96, two were in the higher ALT group. No patients achieved HBsAg seroclearance up to week 96 (Table 2).

HBeAg seroclearance was analyzed in the 69 participants who were HBeAg-positive at baseline. Eight participants (12%) achieved HBeAg seroclearance up to week 48 and 17 participants (25%) achieved HBeAg seroclearance by week 96. HBeAg/anti-HBe seroconversion was analyzed in the 53 participants who were HBeAg-positive and anti-HBe-negative at baseline, and was achieved by seven participants (13%) up to week 48 and 13 participants (25%) by week 96.

The mean (± SD) HBcrAg change from baseline was −0.17 (± 0.29) log_10_ U/mL at week 48 and −0.30 (± 0.36) at week 96. No virologic breakthrough was observed, and serum HBV-DNA levels remained undetectable (< 1.3 log_10_ IU/mL [< 20 IU/mL]) through week 96 in all participants.

### Safety

Adverse events and other key safety analyses are summarized in Table 3.

**Table 3:** Key safety endpoints (SP)

Adverse events (week 96)	TDF (N = 75)
	n (%)
On-therapy AE	58 (77)
On-therapy serious AE	2 (3)
On-therapy AE leading to study withdrawal	1 (1)
Drug-related AE	18 (24)
Drug-related urine beta-2-microglobulin increase	15 (20)
Renal tubular disorder	1 (1)
Renal tubular dysfunction	1 (1)
Prothrombin time prolonged	1 (1)

Other key safety endpoints	n	Median (min, max)	Median change from baseline (min, max)
Serum creatinine (mg/dL)			
Baseline	75	0.70 (0.38, 1.01)	
Week 4	75	0.71 (0.44, 1.02)	0.01 (−0.14, 0.20)
Week 24	74	0.74 (0.41, 1.05)	0.02 (−0.08, 0.15)
Week 48	73	0.72 (0.42, 1.09)	0.04 (−0.11, 0.12)
Week 96	72	0.76 (0.45, 1.02)	0.06 (−0.10, 0.17)
eGFR by JSN-CKDI (mL/min/1.73m^2^)			
Baseline	75	86 (60, 135)	
Week 4	75	84 (60, 138)	−2.0 (−21, 20)
Week 24	74	81 (57, 124)	−3.0 (−21, 13)
Week 48	73	82 (56, 124)	−6.0 (−20, 12)
Week 96	72	79.0 (52, 118)	−9.5 (−24, 14)
Urine beta-2-microglobulin ratio (µg/g creatinine)			
Baseline	75	154.91 (40.71, 1636.09)	
Week 4	75	198.91 (25.72, 4779.66)	45.02 (−317.50, 3143.57)
Week 24	74	185.88 (8.93, 9006.76)	41.17 (−361.52, 7890.23)
Week 48	73	192.50 (13.02, 10,546.26)	38.16 (−582.24, 9429.72)
Week 96	72	154.96 (11.20, 9052.18)	15.19 (−166.80, 7935.65)
Serum phosphorus (mg/dL)			
Baseline	75	3.3 (1.9, 4.4)	
Week 4	75	3.2 (2.0, 4.4)	−0.10 (−1.0, 0.9)
Week 24	74	3.3 (1.4, 4.5)	−0.10 (−1.1, 1.4)
Week 48	73	3.3 (2.0, 4.7)	0.00 (−1.3, 0.9)
Week 96	72	3.4 (2.2, 4.5)	0.10 (−1.1, 1.2)
%TRP			
Baseline	75	89.92 (77.91, 97.71)	
Week 4	75	88.49 (77.04, 98.79)	−0.69 (−13.26, 6.75)
Week 24	74	90.20 (78.96, 96.26)	−0.52 (−11.81, 9.75)
Week 48	73	89.44 (81.51, 97.71)	0.17 (−8.71, 8.27)
Week 96	72	89.83 (81.07, 96.90)	−0.818 (−10.25, 15.47)
ALT (U/L)			
Baseline	75	19.0 (7, 147)	
Week 4	75	22.0 (8, 141)	4.0 (−14, 60)
Week 24	74	22.5 (10, 130)	4.0 (−32, 61)
Week 48	73	23.0 (12, 83)	4.0 (−79, 23)
Week 96	72	21.5 (11, 220)	2.5 (−20, 73)

BMD safety endpoints	n	Median (min, max)	Median % change from baseline (min, max)
BMD, femur (g/cm^2^)			
Baseline	23	0.83 (0.64, 0.99)	
Week 96	23	0.80 (0.66, 1.00)	−2.09 (−12.67, 6.59)
BMD, lumbar vertebra (g/cm^2^)			
Baseline	75	1.00 (0.63, 1.43)	
Week 96	72	0.96 (0.64, 1.52)	−2.24 (−12.21, 6.73)

*AE* adverse event, *ALT* alanine aminotransferase, *BMD* bone mineral density, *eGFR by JSN-CKDI* estimated glomerular filtration rate calculated by the Japanese Society of Nephrology-Chronic Kidney Disease Initiatives (JSN-CKDI) equation, *SD* standard deviation, *SP* safety population, *TDF* tenofovir disoproxil fumarate, *TRP* renal tubular reabsorption of phosphate

Overall, through week 96, most AEs were mild in severity. Two serious AEs, osteoarthritis (one participant) and appendicitis (one participant), were reported but neither was considered to be study drug-related. Drug-related AEs included renal tubular dysfunction and prothrombin time prolonged (one participant each), which were mild in intensity. One participant was withdrawn at the judgment of the investigator due to renal tubular disorder of mild intensity, which resolved. An increase in urine beta-2-microglobulin, considered a sensitive and specific indicator of renal tubular dysfunction associated with TDF [8, 16, 17], was the only renal AE that was reported in more than one participant. An increase of mild intensity occurred in 15 participants (20%) and considered to be study drug-related.

**Fig. 4:**
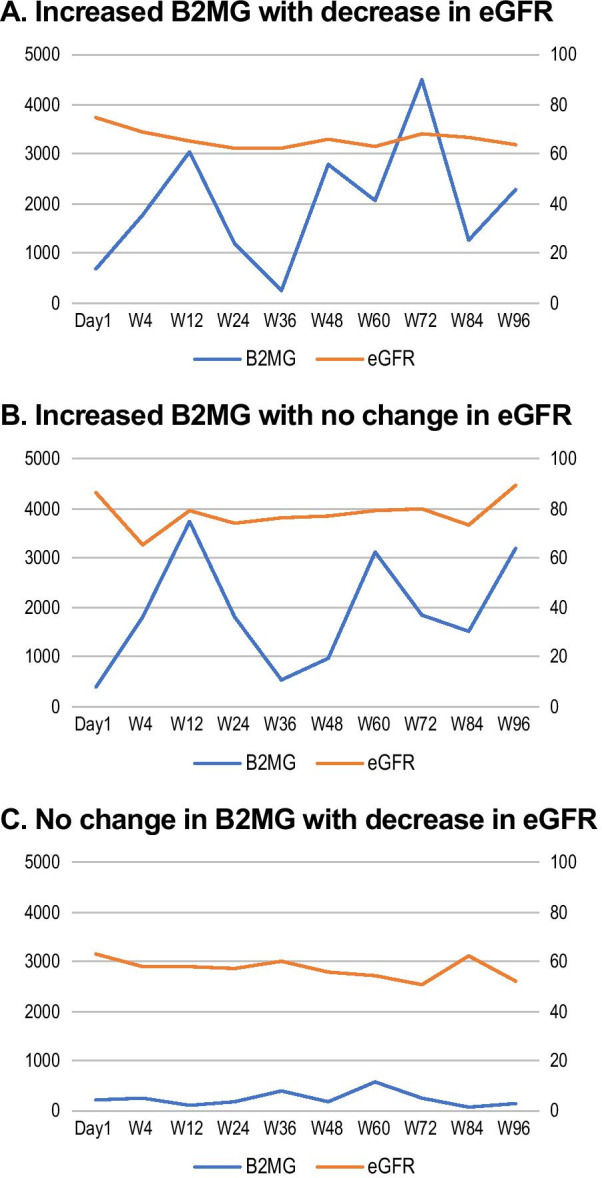
Individual participant observed clinical course types: change in B2MG (µg/g creatinine) and eGFR (mL/min/1.73m^2^). B2MG, urine beta-2-microglobulin; Cr, creatinine; eGFR, estimated glomerular filtration rate

In addition to the increase in median urine beta-2-microglobulin (median change from baseline 15.19 µg/g creatinine), a small increase in median serum creatinine (0.06 mg/dL) and a decrease in median estimated glomerular filtration rate (eGFR, −9.5 mL/min/1.73m^2^) were observed at week 96. Not all participants with an increase in urine beta-2-microglobulin experienced a decrease in eGFR, and there were no decreases in median percent renal tubular reabsorption of phosphate (%TRP) or serum phosphorus except for two participants who experienced hypophosphatemia, which were not considered to be study drug-related. Overall, there were three types of clinical courses observed amongst participants who experienced a change in renal parameters (Fig. 4): 1) participants with an increase in urine beta-2-microglobulin and decrease in eGFR (Fig. 4a), 2) those with an increase in urine beta-2-microglobulin and without change in eGFR (Fig. 4b), and 3) those without a change in urine beta-2-microglobulin and decrease in eGFR (Fig. 4c).

No participants had drug-related liver AEs. The only liver AESI was hepatic steatosis, not drug related, reported by one participant. No clinically significant elevation of ALT was observed (Table 3). At baseline, six participants (8%) had ALT levels above the upper limit of normal (> 40 U/L) compared with 10 participants (14%) at week 48 and five (7%) at week 96.

No participants had drug-related bone AEs or AESIs. The only bone AESI was jaw pain, not drug related, reported by one participant. A decrease in bone mineral density (BMD) (median change from baseline: femur, −2.09%; lumbar vertebra, −2.24%) was observed at 96 weeks.

## Discussion

This study is the first prospective study to investigate the efficacy and safety of switching to TDF from ETV, focusing on patients with HBeAg-positive and HBV-DNA undetectable CHB in Japan. Because the results of our previous phase 3 study in treatment-naïve participants showed that reduction in HBsAg observed in the TDF group was predominantly in HBeAg-positive patients [13], we targeted patients with HBeAg-positive CHB (including five patients who were HBeAg-positive according to local in-hospital laboratory results; Table 1) treated with ETV for at least two years in this phase 4 study. To ensure an adequate sample size of HBeAg positive CHB patients with undetectable serum HBV-DNA, the study was conducted as a multi-center trial.

While our data showed the switch to TDF was still virologically suppressive, the proportion of patients achieving 0.25 log_10_ HBsAg reduction from baseline at week 48 (4%) was lower than our original assumption (20%). This may be explained by the variation in duration of previous ETV treatment across the study population. Entecavir therapy for at least two years prior to screening was required, however, no upper limit was specified for duration of time on therapy and participant data were not collected in this regard. Previous studies have reported a greater rate of HBsAg reduction during the first year of NA therapy (ETV, TDF) compared with subsequent years [18]. Although we did not investigate the correlation between the duration of ETV therapy and HBsAg reduction in this study, it is possible that the duration of prior ETV therapy influenced the rate of patient achieving 0.25 log_10_ HBsAg reduction from baseline at week 48.

Participants in this study with higher ALT levels (N = 6, defined as ≥ 60 U/L at least once from baseline through week 48), including the three participants with 0.25 log_10_ HBsAg reduction from baseline at week 48, had a numerically greater reduction from baseline in HBsAg level at both week 48 and week 96 versus those with lower ALT levels (N = 67, defined as < 60 U/L from baseline through week 48). This is similar to our previous study in naïve patients indicating that HBsAg reduction was predominant in patients with higher ALT levels (≥ 80 U/L) [13]. However, in terms of individual participants, there was no apparent pattern between ALT levels and HBsAg change from baseline for the nine participants in the current study who achieved > 0.25 log_10_ reduction at 96 weeks. It is possible that a combination of factors (e.g., high ALT together with HBeAg-positive status and some unknown factors) is associated with HBsAg reduction after switching to TDF. As previously reported, higher serum IFN λ3 levels were observed in patients treated with NAs (ADV and TDF), but not in those treated with NAs (lamivudine and ETV) [19]. Interferon-λ3 induction caused by the NAs further induces IFN-stimulated genes and results in a reduction of HBsAg production. Although we did not measure serum IFN λ3 levels in this study, greater HBsAg reduction in patients with higher ALT levels may be related to immunomodulatory effects associated with TDF therapy [20].

In this study, 25% of HBeAg-positive participants, with at least two years of prior ETV treatment, achieved HBeAg seroclearance up to 96 weeks after switching to TDF. The HBeAg seroclearance rate we observed was comparable to rates reported in previous studies in patients treated with ETV [21, 22]. In this limited number of participants, there was no apparent relationship between HBeAg status change and HBcrAg reduction.

No new safety concerns were identified with TDF therapy (including bone, liver, or renal AESIs) compared with previous studies [5, 8, 11, 13, 14]. Among participants with a decrease in BMD, no apparent common trends were observed, such as a reduction in serum phosphate or %TRP, and there were no apparent associated common factors. Overall, decreases in BMD were consistent with historical data for participants with HBV and the known safety profile of TDF therapy [23]. There was no apparent link between changes in BMD and other renal parameters measured in the current study. Observed increases in serum creatinine and decreases in eGFR were also comparable to previous studies [8, 11, 14], and therefore, continuous monitoring for bone and renal events is necessary during treatment with TDF.

The percentage of participants with ALT above the upper limit of normal (> 40 U/L) increased to 14% at week 48 from 8% at baseline, although it is unclear if the lack of ALT normalization was due to TDF therapy [11]. We also carefully monitored urine beta-2-microglobulin to creatinine ratio and %TRP. Although urine beta-2-microglobulin to creatinine ratio and %TRP are considered sensitive and specific indicators of renal tubular dysfunction associated with long-term use of TDF [8, 16], no consistent trends were apparent in the current study between changes in these early indicators of kidney injury and other renal parameters such as eGFR. An increase in urine beta-2-microglobulin was not always accompanied by a change in eGFR and some participants had a decrease in eGFR with no change in urine beta-2-microglobulin. These observations suggest that urine beta-2-microglobulin may not be a sensitive marker for predicting renal dysfunction.

This study has several limitations that should be considered. In previous studies, HBsAg levels have been shown to decline with ETV treatment [21, 22, 24, 25]. Because there was no control arm in this study due to feasibility constraints, it was not clearly shown that the HBsAg decline (4% responder rate, mean 0.11 log_10_ IU/mL decline from baseline) observed at week 48 was attributed to TDF switch therapy. Another limitation is that most of the patients in the current study had genotype C, whereas HBsAg loss occurs more often and declines in HBsAg are greater in HBeAg + patients with genotype A or D infection [4]. Finally, patients with clinically significant renal impairment (creatinine clearance < 70 mL/min) were not assessed, which could have biased renal outcomes in this study.

## Conclusions

Although switching from ETV to TDF remained virologically suppressive with no significant safety concerns, our analysis did not clearly show an additional clinical benefit of switching from ETV to TDF in HBeAg-positive and HBV-DNA undetectable CHB patients. Nevertheless, our analysis suggests that there are additional patient factors impacting HBsAg reduction. Evaluation of liver damage and its reversibility with CHB treatment are important clinical questions that were not addressed in the current study which was focused on virological response. Further investigation is needed to better understand the clinical impact of switching from ETV to TDF. Our study also indicates a need for further development of HBV therapies with a novel mechanism for HBsAg reduction and seroclearance.

## Data Availability

The datasets used and/or analyzed during the current study can be requested for further research. Within 6 months of this publication, anonymized individual participant data, the annotated case report form, protocol, reporting and analysis plan, data set specifications, raw dataset, analysis-ready dataset, and clinical study report will be available for research proposals approved by an independent review committee. Proposals should be submitted to www.clinicalstudydatarequest.com. A data access agreement will be required.

## Abbreviations

ADV: Adefovir dipivoxil
AE: Adverse event
AESI: Adverse event of special interest
ALT: Alanine aminotransferase
BMD: Bone mineral density
CHB: Chronic hepatitis B
eGFR: Estimated glomerular filtration rate
ETV: Entecavir
HBcrAg: Hepatitis B core-related antigen
FAS: Full analysis set
HBeAg: Hepatitis B e-antigen
HBsAg: Hepatitis B surface antigen
HBV: Hepatitis B virus
HCV: Hepatitis C virus
HCC: Hepatocellular carcinoma
IFN: Interferon
NA: Nucleos(t)ide analogue
TAF: Tenofovir alafenamide
TDF: Tenofovir disoproxil fumarate
TRP: Renal tubular reabsorption of phosphate
